# Tongue Mucoceles: a retrospective clinic-pathological evaluation of 240 cases

**DOI:** 10.1186/s12903-023-03485-y

**Published:** 2023-11-14

**Authors:** Romeo Patini, Michele Giuliani, Gioele Gioco, Mariateresa Tranfa, Vito Carlo Alberto Caponio, John Fantasia, Carlo Lajolo

**Affiliations:** 1 Department of Head, Neck and Sense Organs, School of Dentistry, Catholic University of Sacred Heart, Rome, Italy; 2 Department of Clinical and Experimental Medicine, University of Foggia, Foggia, Italy; 3 Dentistry - IRCCS “Casa Sollievo della Sofferenza” San Giovanni Rotondo, Foggia, Italy; 4 Long Island Jewish Medical Center, Hofstra University – LIJMC, Hempstead, NY USA

**Keywords:** Tongue, Mucocele, Retrospective study

## Abstract

**Background:**

Minor salivary glands can be found in the ventral and anterior part of the tongue; these glands can rarely develop mucoceles that, due to their rarity and their unusual clinical appearance, may present an interesting differential diagnosis. Mucoceles appear as an exophytic, sometimes pedunculated, lesion, which is a feature that is due to the absence of a capsule; thus, the glands are right beneath the mucosa and over the muscle tissue. The aim of this article is to retrospectively present and discuss the anatomy, pathology, clinical features and therapy of several cases of Blandin-Nunh mucoceles collected from two different institutions.

**Methods:**

A retrospective case review was carried out in two university institutions, retrieving all cases of tongue mucoceles from 1999 to today. Two oral pathologists reviewed all the slides, confirming the diagnosis. Demographic data of the patient, anatomic location and clinical appearance were retrieved from clinical charts, together with the type of surgical procedure and possible relapses.

**Results:**

A total of 240 cases of tongue mucoceles were gathered from the archives: the mean age was 22 years (DS = 14,7; Range 2–83), 126 were females (52,5%, mean age 22,7 years, DS = 16,5; Range 2–83), and 114 were males (47,5%, mean age 20,9 years, DS = 12,4; Range 3–73); in all cases, a history of trauma was reported. The ventral surface was the most frequent location (224 cases – 93,3%), and in the great majority (235 cases – 97,9%), pathology revealed mucous spillage with a wall formed by fibrous connective and granulation tissue with no epithelium lining the cavity. Superficial mucocele and sclerosing sialoadenitis were the more frequent pathological variants (21 cases – 8,8%). All lesions were treated with excision and enucleation of the servicing gland. The healing was uneventful in all cases, but there were four recurrences and two cases of sensory paraesthesia of the border of the tongue, all in males, except one case of paraesthesia in a female.

**Conclusions:**

Tongue mucoceles must be differentiated from many benign and malignant lesions. For this reason, surgical removal of the lesion and of the associated gland with a pathological exam is mandatory. In fact, the anatomical location of the glands and the possible pathological variants must be considered to reach a correct diagnosis and diminish possible relapses.

**Trial registration:**

CE-Muc_Ton_3/2023.

**Supplementary Information:**

The online version contains supplementary material available at 10.1186/s12903-023-03485-y.

## Introduction

Among the minor salivary glands of the oral cavity, three major groups have been described within the tongue: Weber glands (mainly mucous) located on the border of the tongue and in the crypt of the lingual tonsils; von Ebner glands (mainly serous), which are located within circumvallate papillae and seem to play a fundamental role in gustative sensibility; and Blandin-Nuhn glands, which are mixed (serous and mucous) glands located in the ventral surface of the tongue. Blandin-Nuhn glands are larger than the other two subsets of glands; they are 8 × 15 mm wide, located 12–25 mm deeply within the tongue muscles, slightly laterally to the median line, and medially to the plica fimbriata (fimbriated fold of the tongue), and covered by a thin mucosa [1–3].

Some epidemiological studies carried out on a large number of mucoceles showed that Blandin-Nuhn mucoceles (BNMs) represent 2 to 15% of minor salivary gland mucoceles depending on the studied Groups [4–7], whereas to the best of our knowledge, von Ebner mucoceles were described in the literature in only two case reports [8, 9], and mucoceles of Weber glands were never described. Literature reports showed that BNMs present a youth prevalence (within the fourth decade) and a female predilection [4–7]. The etiopathogenesis of BNMs seems to be related to mucous retention or extravasation caused by a previous trauma (bite) or complications of oral surgery or can be congenital [3–5].

**Fig. 1:**
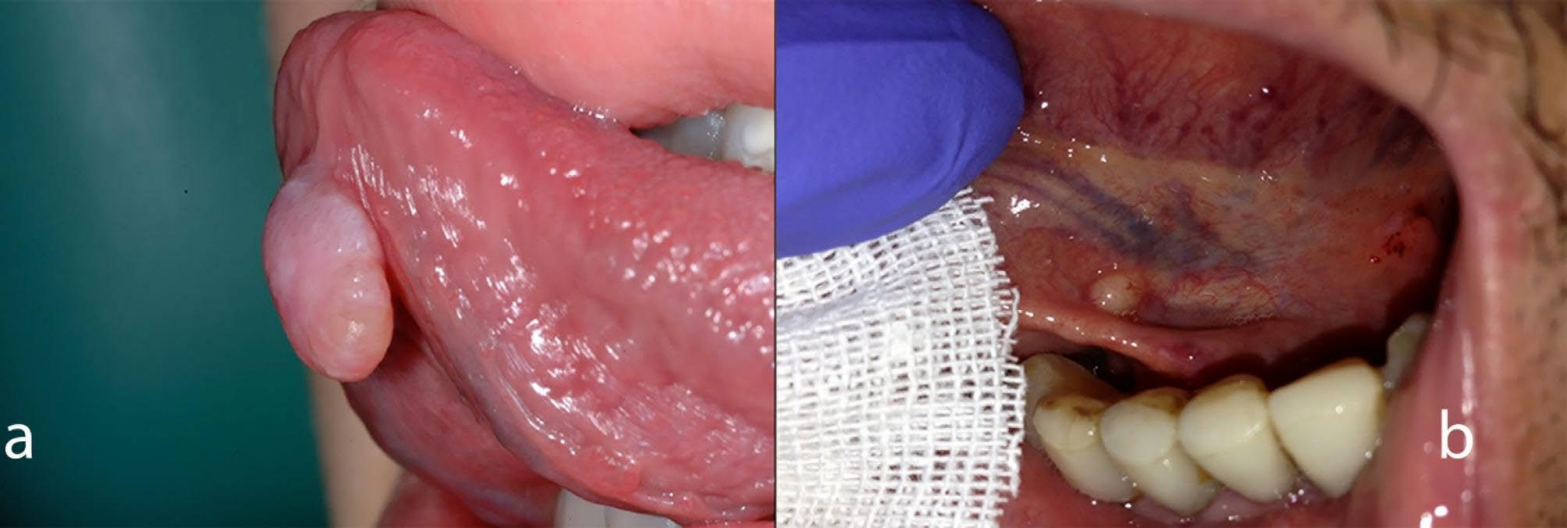
Two clinical presentations can usually be seen for BNMs

Two clinical presentations can usually be seen for BNMs: an exophytic lesion, either sessile or pedunculated, light pink in colour, with a tensile/elastic consistency, one centimetre in diameter, and painless with some areas of possible ulceration due to trauma; the second presentation is a lesion that is round, small (less than one centimetre), translucent, and right beneath the mucosa, with yellowish raised swelling, and the lesion is slightly floating, movable on the deeper layers, and painless (Fig. 1) [3]. Other clinical presentations have been described as being particularly large and life-threatening [10–12].

Because these lesions are quite rare and present with unusual clinical features, the diagnosis of tongue mucoceles can be difficult.

The aim of this article is to retrospectively present and discuss the anatomy, pathology, clinical features and therapy of several cases of Blandin-Nunh mucoceles collected from two different institutions.

## Materials and methods

A retrospective case review was carried out in two university institutions (Long Island Jewish Medical Center, Hofstra University – LIJMC, NY, USA and Università Cattolica del Sacro Cuore, Rome, Italy) retrieving all cases of tongue mucoceles from 1999 to today. The retrospective study was approved by the ethics committee (CE-Muc_Ton_3/2023). The study was conducted according to the Declaration of Helsinki, and all patients signed an informed consent form.

Two oral pathologists reviewed all the slides, and the presence of mucin spillage (H&E or mucicarmine) with an associated inflamed granulation tissue response or cystic wall was used for pathology diagnosis.

Patient demographic data (age and sex), anatomic location, and clinical appearance were retrieved from clinical charts together with the surgical procedure, possible relapses and complications. Mucoceles arising in other anatomic sites (i.e., floor of the mouth - ranula) were excluded.

### Statistical analysis

Descriptive statistics were calculated as the means and standard deviations if they presented with a normal distribution. Discontinuous variables were calculated as frequencies and percentages. Statistical analysis was performed using the software program “Intercooled Stata”, version 8.0 (Stata Corp, College Station, Texas, USA).

## Results

A total of 248 cases were gathered from the archives, 8 of which were excluded (i.e., not tongue, not mucoceles, incomplete documentation), so a final number of 240 cases of tongue mucoceles were selected. The mean age was 22 years (sd = 14,7; Range 2–83), 126 were females (52,5%, mean age 22,7 years, sd = 16,5; Range 2–83), and 114 were males (47,5%, mean age 20,9 years, sd = 12,4; Range 3–73). the age distribution of the sample is summarized in Table 1. In all cases, a history of trauma was reported. The ventral surface was the most frequent location (224 cases – 93,3%), and in the great majority (235 cases – 97,9%), pathology revealed mucous spillage with a wall formed by fibrous connective and granulation tissue with no epithelium lining the cavity. Superficial mucocele and sclerosing sialoadenitis were the more frequent pathological variants (21 cases – 8,8%). The location, pathology and the pathological variants are summarized in Table 2.

**Table 1:** Age distribution of the sample

	Total population		Females		Males	
Age (years)	Frequency	Percent	Frequency	Percent	Frequency	Percent
0–5	5	2.1	2	1.6	3	2.6
5–10	39	16.2	21	16.7	18	15.8
10–20	110	45.8	59	46.8	51	44.7
20–30	37	15.4	18	14.3	19	16.7
30–40	22	9.2	8	6.3	14	12.3
40–50	14	5.8	7	5.6	7	6.1
> 50	13	5.4	11	8.7	2	1.8

**Table 2:** Clinical and pathological characteristics of the samples

	Total population		Females		Males	
	N°	%	N°	%	N°	%
	240		126	52.5	114	47.5
**Location**						
Ventral surface (between root and tip)	224	93.3	117	92.9	107	93.9
Tip	7	2.9	4	3.2	3	2.6
Lateral surface	4	1.7	3	2.4	1	0.9
Root	3	1.2	1	0.8	2	1.8
Other (dorsum)	2	0.8	1	0.8	1	0.9
**Pathological finding**						
Extravasation type	235	97.9	122	96.8	113	99.1
Cystic type	5	2.1	4	3.2	1	0.9
**Variants**						
Sclerosing sialoadenitis	21	8.8	12	9.5	9	7.9
Superficial mucocele	21	8.8	13	10.3	8	7.0
Ulcerated mucosa	5	2.1	2	1.6	3	2.6
Hyperparakeratosis of the surrounding (adjacent) mucosa	2	0.8	1	0.8	1	0.9
Vascular component dilatation	2	0.8	1	0.8	1	0.9
Associated Lymphoid tissue	1	0.4	1	0.8		
Myxoglobulosis (collagenous spherulosis)	1	0.4	1	0.8		

All lesions were treated under local anaesthesia (standard block and infiltrative manner, using 2% carbocaine with 1:100,000 epinephrine) and with excision and enucleation of the servicing gland (dissection) by 3 oral surgeons with at least 10 years of practice; the wound was sutured with 3–4 or 4 − 0 silk suture; and no antibiotic was prescribed unless the patient was suffering from systemic disease (i.e., diabetes).

The healing was uneventful in all cases, but there were four recurrences and two cases of sensory paraesthesia of the border of the tongue, all in males, except one case of paraesthesia in a female.

## Discussion

Even if some authors state that Blandin-Nuhn mucoceles are rare, this study reports 240 cases of Blandin-Nunh mucoceles collected from two different institutions, thus representing the largest case series presented in the literature. The available epidemiological studies conducted on oral mucoceles showed a 15 to 18% frequency, being second only to lower lip mucoceles [4–7]. In fact, our study confirms that tongue mucoceles have a greater incidence in young people, 50% of them presenting before 20 years of age and with a peak between 10 and 20 years; nonetheless, some cases can occur in infants [13] and in elderly people [14]. In our individual patient data analysis, sex did not appear to be a risk factor, even if there was a slight predilection for female sex.

**Fig. 2:**
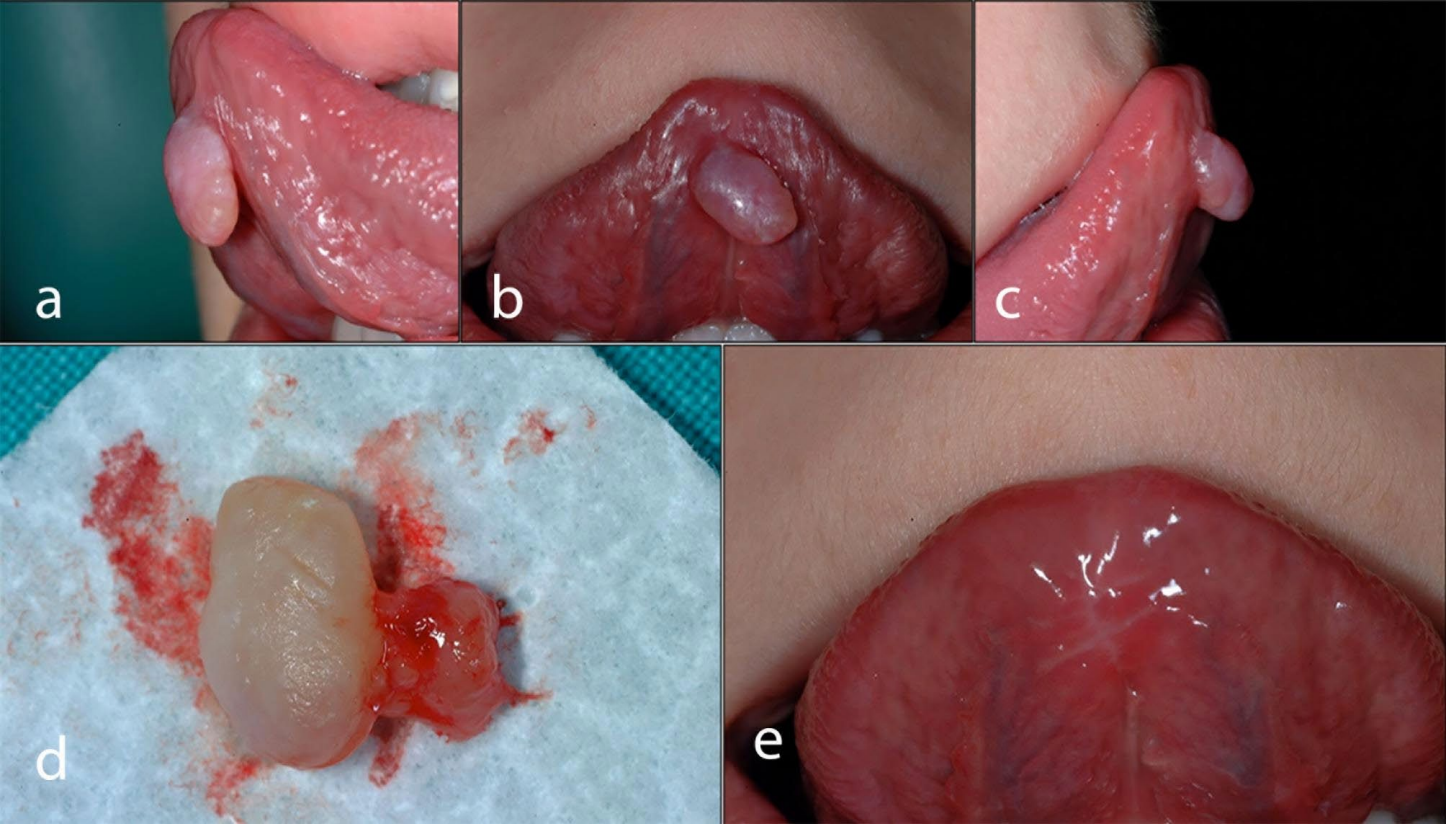
Oral presentation for BNMs and after surgical excision

Tongue trauma (a bite wound or repeated trauma against the lower incisal border) was reported in all our cases as the main aetiological factor: in fact, pathology showed the presence of extravasation mucoceles in more than 95% of the cases (mucous spillage surrounded by connective and granulation tissue - Fig. 2) and, more rarely, the presence of a real cyst (mucous plug/calculus). Extravasation mucoceles are due to the breakdown of the excretory duct, which is usually from being traumatized: such extravasation of mucous into the connective tissue causes a subsequent foreign body reaction (macrophages and neutrophils migration - diapedesis) and the formation of pseudocysts. Moreover, some authors postulated that young people have a higher risk of developing mucoceles because of the sharp margins of the lower incisors, from possible parafunctional habits and, more frequently, from oscillation of the tongue [1–5].

Considering the clinical presentation, more than 90% of cases (224) involved the ventral surface of the tongue, and among these, the majority (209–93,3%) presented the typical clinical features of extravasation mucoceles (exophytic lesion, pedunculated, light pink in colour, with a tensile/elastic consistency, one centimetre in diameter, without pain, some areas of possible ulceration due to trauma); thus, we confirm the previous literature reports [2]. In some cases, especially at the onset of swelling, the lesion can be bluish in colour due to the extravasation of blood from the involved injured capillaries. The remaining cases presented the features of superficial raised mucoceles (small, i.e., ≤ 5 mm, rounded clear/translucent vesicles) [3]. In our series, some other tongue localizations were detected: 4 of them were on the lateral surface (border of the tongue).

**Fig. 3:**
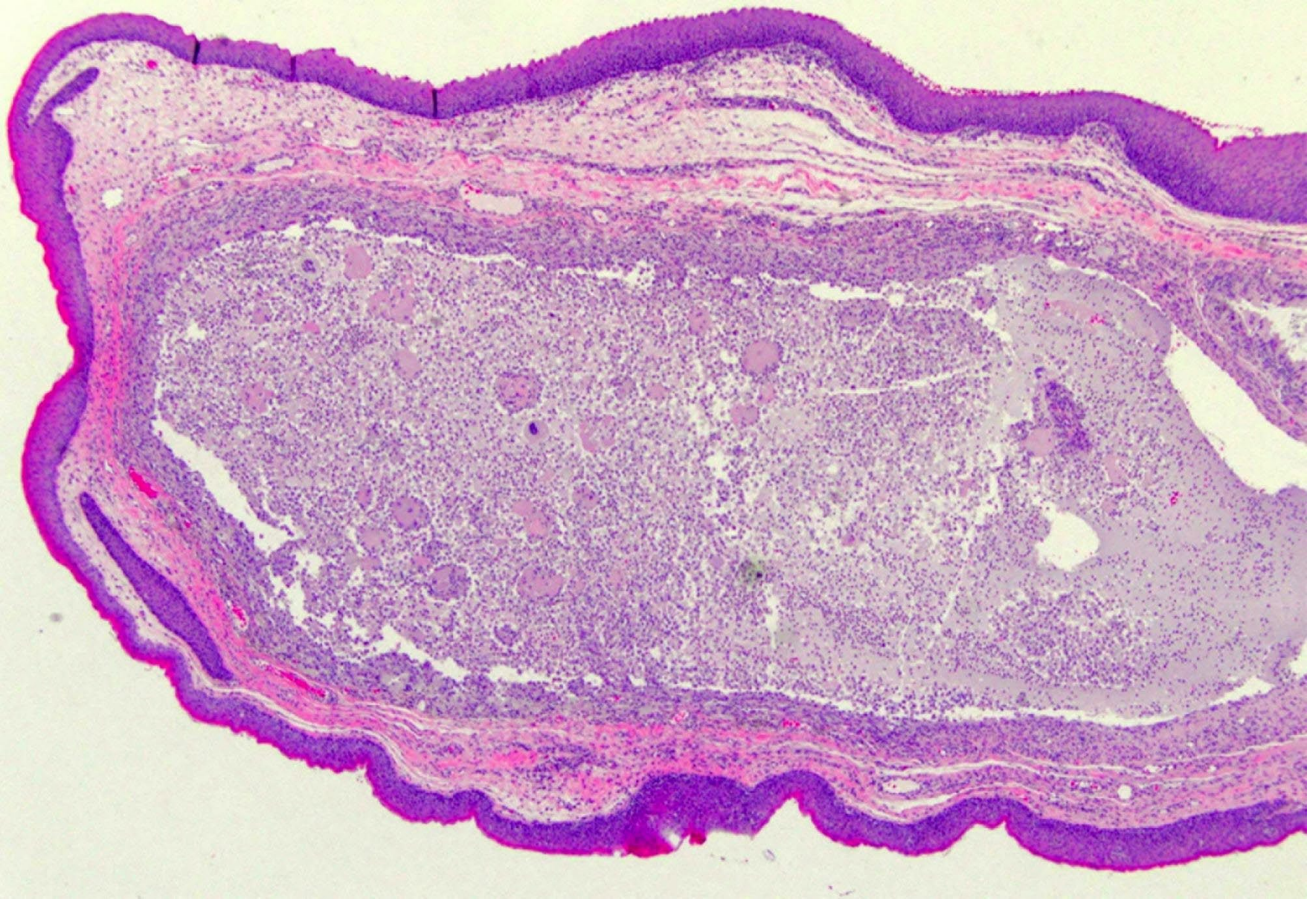
Pathological characteristics of BNM.

The great majority of our cases presented mucous spillage within a circumscribed cavity in the connective tissue of the submucosa (extravasation-type – 97%), with no epithelium lining the cavity and walls formed by fibrous connective tissue and granulation tissue (foreign body inflammatory reaction - Fig. 3). In a few cases, a real cyst, with epithelium lining the cavity due to a mucous plug or stone, was detected.

Considering the variants described in the literature, ulcerated mucosa (2%), superficial mucocele (8,8%) and sclerosing sialadenitis (8,8%) were the most frequently identified variables in our series, and only one case of myxoglobulosis or “collagenous spherulosis” (mucinous globular structures) was also found. No cases of papillary synovial metaplasia-like change were identified. Nevertheless, it is important for pathologists to be aware of these rare variants to avoid a misdiagnosis.

Superficial mucocele may potentially be confused with immune-mediated subepithelial vesiculobullous conditions (such as pemphigoid or bullous lichen planus) or the vesicles of recurrent herpes simplex virus infection [15]. Their microscopic features are as follows: subepithelial blister containing mucin; often attenuated surface epithelium; possible regeneration of the epithelium across the floor of the blister; and no evidence of extension of subepithelial separation at the periphery of the lesion. Therefore, it is quite simple to understand that such clinical and microscopic features may be mistaken for other conditions, such as the abovementioned pemphigoid, bullous lichen planus or recurrent HSV infection.

Papillary synovial metaplasia-like change is defined by the following criteria: (1) synovial-like membranes thrown into papillary folds and (2) the membrane surface included a thin band of acellular eosinophilic matrix with an underlying condensation of palisaded histiocytes, fibrohistiocytes and/or multinucleated giant cells [16]. Papillary synovial metaplasia-like changes should be distinguished from salivary neoplasms with a papillary cystic growth pattern (such as oncocytic papillary cystadenoma or Warthin tumour). In these cases, the typical mucocele granulation tissue wall was partially replaced by a membrane exhibiting villous folds, a thin superficial band of eosinophilic matrix, and an underlying condensation of palisaded histiocytes, fibrohistiocytes, and/or multinucleated giant cells. Synovial metaplasia is typically seen in other settings, such as in association with breast implants, the bone-cement interface of hip prostheses, tendon implants, testicular implants, and traumatized skin. We believe these changes represent a response to trauma and only rarely arise within oral mucoceles.

Myxoglobulosis, instead, is defined as the presence of hyalinized globular structures either free within the lumen or attached to the granulation tissue wall [17]. However, in the literature, it is reported that such changes often represent a response to trauma and only rarely arise within oral mucoceles.

Considering the diagnostic protocol and differential diagnosis, great value should be given to the history of a rapid onset swelling arising after a trauma (usually a bite), which tends to recur with rapid changes: the swelling, initially floating at the beginning and easily movable, tends to become adherent to the underlying layers. In some cases, at the onset, the lesion may appear bluish in colour, but after some recurrences, it becomes light pink. Among benign and malignant lesions, which can arise on the ventral tongue, HPV-related lesions, pyogenic granulomas, vascular lesions and oral cancer must be considered as differential diagnosis. A fine needle aspiration biopsy (FNAB) can be helpful in the diagnostic workup to exclude other diseases [18], but the correct diagnosis can be reached only with a biopsy. In fact, the presence of a mucous spillage surrounded by connective and granulation tissue (i.e., pseudocysts) or a mucous collection within an epithelial wall is enough to confirm the clinical hypothesis.

Among the different therapeutic approaches, excision, marsupialization, dissection (i.e., excision and enucleation of the gland), cryosurgery, laser ablation and medical therapy have all been described [3, 6, 19–22]. Careful clinical evaluation of these lesions and preoperative awareness of the surgical anatomy of the glands may minimize the risk of relapses; the anatomical location of the glands, especially Blandin-Nuhn glands, which are located deep into the muscular tissue, must be clearly taken in mind when planning the enucleation of the servicing mucous glands. To diminish the possibility of relapses after excision, the servicing gland must be removed up to the muscular plane, as performed in all the mucoceles removed in our study. An additional movie file shows this in more detail (see Additional file 1). Nevertheless, we had 4 relapses that occurred in males and one sensory paraesthesia of the border of the tongue. Considering the other therapeutic approaches, some interest must be considered for marsupialization of the lesion, which can be helpful to resolve large mucoceles, or in paediatric patients before performing definitive surgical enucleation [19]. Another proposed approach is the administration of intralesional steroids, which has been advocated as a possible alternative therapy when clinicians decide to avoid surgery [23]. Recently, moreover, a case report [24] and a clinical trial [25] showed that laser can represent a valid substitute for the cold scalpel in the surgical management of mucoceles or in promoting the healing of mucocele marsupialization [26], but the majority of the treated lesions were in the lower lip, thus with superficial servicing glands. For these reasons, traditional surgical excision with enucleation of the deep servicing gland seems to be the most reliable therapeutic approach.

## Conclusions

Tongue mucoceles must be differentiated from many benign and malignant lesions (HPV-related lesions, pyogenic granulomas, vascular lesions and oral cancer) that may arise on the tongue. Surgical removal of the lesion and of the gland with a pathological exam is mandatory; the anatomical location of the glands (i.e., Blandin Blandin–Nuhn are located deep in the muscles of the tongue) and possible pathological variants must be considered to reach a correct diagnosis and diminish the possible relapses.

### Electronic supplementary material

Below is the link to the electronic supplementary material.

Supplementary Material 1

## Data Availability

The datasets used and/or analysed during the current study are available from the corresponding author upon reasonable request.

## List of abbreviations

BNMs: Blandin-Nuhn Mucoceles
H&E: haematoxylin and eosin
HSV: herpes simplex virus
HPV: human papillomavirus
FNAB: fine needle aspiration biopsy
